# Analysis of repetitive amino acid motifs reveals the essential features of spider dragline silk proteins

**DOI:** 10.1371/journal.pone.0183397

**Published:** 2017-08-23

**Authors:** Ali D. Malay, Kazuharu Arakawa, Keiji Numata

**Affiliations:** 1 Enzyme Research Team, Center for Sustainable Resource Science, RIKEN, Wako-shi, Saitama, Japan; 2 Institute for Advanced Biosciences, Keio University, Kakuganji, Tsuruoka, Yamagata, Japan; Tianjin University, CHINA

## Abstract

The extraordinary mechanical properties of spider dragline silk are dependent on the highly repetitive sequences of the component proteins, major ampullate spidroin 1 and 2 (MaSp2 and MaSp2). MaSp sequences are dominated by repetitive modules composed of short amino acid motifs; however, the patterns of motif conservation through evolution and their relevance to silk characteristics are not well understood. We performed a systematic analysis of MaSp sequences encompassing infraorder Araneomorphae based on the conservation of explicitly defined motifs, with the aim of elucidating the essential elements of MaSp1 and MaSp2. The results show that the GGY motif is nearly ubiquitous in the two types of MaSp, while MaSp2 is invariably associated with GP and di-glutamine (QQ) motifs. Further analysis revealed an extended MaSp2 consensus sequence in family Araneidae, with implications for the classification of the archetypal spidroins ADF3 and ADF4. Additionally, the analysis of RNA-seq data showed the expression of a set of distinct MaSp-like variants in genus *Tetragnatha*. Finally, an apparent association was uncovered between web architecture and the abundance of GP, QQ, and GGY motifs in MaSp2, which suggests a co-expansion of these motifs in response to the evolution of spiders' prey capture strategy.

## Introduction

Spiders produce multiple types of silk to fulfill a variety of biological tasks, including web construction, prey wrapping, and protection of eggs [1]. Among the variety of spider silks, dragline silk (major ampullate silk) has attracted the most attention because of its relative ease of reeling, and due to its truly outstanding mechanical properties, which can surpass the most sophisticated man-made fibers [2]. Spiders use dragline silk as a safety line and as a major component of webs, and constitutes the frame and main radii of orb-type webs. The extreme strength and toughness of dragline silk are critical for the absorption and dissipation of the kinetic energy imparted by swiftly flying insects on the web structures [2, 3].

Spider silks are composed primarily of spidroins, structural proteins whose architecture usually consists of a long, repetitive central domain flanked by conserved globular amino- and carboxyl-terminal domains (NTD and CTD) [4]. The different spider silks are associated with distinct types of spidroins that are encoded by different members of a gene family [5]. Dragline silk is remarkable for harboring two spidroin components, the major ampullate spidroins 1 and 2 (MaSp1 and MaSp2) [4, 6, 7]. The genes encoding MaSp1 and MaSp2 share a complicated evolutionary history that indicates past duplication and recombination events, and in some cases show evidence for multiple functional loci [8–12]. In both MaSp proteins, the bulk of the sequence (>90%) is taken up by the central repetitive domain, which is organized as numerous modules (tandem repeats) of alternating polyalanine (poly-Ala) and glycine-rich (Gly-rich) regions. The Gly-rich regions are arranged in arrays of concatenated amino acid motifs dominated by GX and GGX, with X corresponding to a subset of residues with high interspecific variability. During the spinning process, the poly-Ala regions form intermolecular β-sheets that constitute the crystalline fraction of the resultant fiber, while the Gly-rich regions mostly make up the so-called amorphous matrix [13, 14]. MaSp1 and MaSp2 sequences are generally differentiated based on conserved proline residues in the latter's tandem repeats, often in the context of iterated GPGXX runs [6, 9, 13]. Variations in MaSp1/MaSp2 ratios, as found across phylogenetic groups and between individuals of the same species, are thought to play an important role in modulating the mechanical properties of dragline silk [15–17].

An intimate relationship links the physical properties of silk to the primary structure of the underlying protein components [4, 18]. Dragline spidroins from different spider taxa display highly diverse amino acid motif repertoires, and these variations are believed to exert a considerable impact on the mechanical properties by contributing mainly to secondary structural variations within the silk fiber [4, 13]. The poly-Ala regions, along with flanking (GA)_n_ elements, adopt β-sheet structures that make up the nano-crystalline fraction that is responsible for the impressive strength of silk fiber [14, 19], while GGX motifs have been associated with 3_1_-helical conformations in the amorphous matrix [20]. In MaSp2, the proline-rich sections of the Gly-rich regions are thought to adopt β-turn conformations that contribute to the high mobility of the polypeptide chains [6, 13, 21, 22]. Importantly, a correlation has been found between the abundance of proline residues in MaSp2 repetitive domains and the degree of elasticity and supercontraction of the corresponding dragline fiber [17, 23–26]. Apart from these examples, however, there have been few studies that link specific motif types (or their arrangements) to particular biomolecular functions or physical properties of dragline silk. A deeper exploration of these motif-structure-function relationships could help elucidate some of the fundamental, yet poorly understood aspects of dragline silk, such as regarding the hierarchical organization of the spidroin components in the gland and in the fiber [27, 28] or the molecular basis for water-induced supercontraction [29, 30], among others [31]. Insights gained from such studies could also lead to significant advances in the design of recombinant proteins toward the production of artificial dragline silks with biomimetic properties, a much sought-after goal in bioengineering that has so far not been achieved [31, 32].

In this study, we performed a systematic analysis of the available MaSp sequences, with the primary aim of elucidating the essential motif elements in MaSp1 and MaSp2 tandem repeat sequences based on empirical criteria. Secondly, we sought to uncover novel patterns of tandem repeat organization, as well as probe the relationships between conserved motif patterns and other aspects of spider biology. Our approach differs from previous analyses by the implicit treatment of the different amino acid motifs as discrete units of selection, whereas other studies tend toward more generalized analyses (and typically include non-MaSp sequences in the analysis). In addition, the scope of the sequences included is considerably broader than in previous investigations: all available MaSp (and MaSp-like) sequences were surveyed, taking advantage of the large number of data currently found in the databases, and thus ensuring a wide coverage of spider taxa; in addition, recent insights from spider systematics were incorporated into the analysis [33, 34]. The study is presented as interrelated subsections, each with a different area of focus: (1) identification of conserved amino acid motifs via the analysis of reference MaSp sequences; (2) validation of the resultant consensus motif profiles by screening MaSp sequences from GenBank; (3) analysis of serial motif organization patterns from family Araneidae; (4) evaluation of divergent sequences (from *Tetragnatha*) through the use of supplementary RNA-seq data; and (5) analysis of conserved motif abundance as a function of spiders' web building behavior. Starting from a huge diversity of sequences, our motif-based analysis identified a small subset of conserved motif elements associated with MaSp1 and MaSp2 tandem repeats, spanning the Entelegynae clade. Particularly, in MaSp2 sequences the prevalence of 3 motifs GP, QQ, and GGY were found to vary as a function of spider web morphology, and are hypothesized to cooperatively modulate the mechanical properties of dragline silk.

## Methods

### Motif-based analysis of MaSp1 and MaSp2 sequences

The consensus motif profiles for MaSp1 and MaSp2 were constructed by analyzing the sequences from the following 5 reference spider species, *Latrodectus hesperus*, *Nephila clavipes*, *Araneus diadematus*, *Argiope bruennichi*, and *Euprosthenops australis*; the relevant GenBank accession codes are listed in S1 Table. The tandem repeats within each sequence were internally aligned by eye using Geneious v.9 software, and conserved amino acid motifs were detected to thus generate sequence-specific profiles. In particular, the presence of short, 2- or 3- residue motifs corresponding to the patterns GX or GGX (where X corresponds to A, S, Y, Q, D, R, P, N, L, or F), as well as QQ and SS, were detected. The GX and GGX patterns were treated as mutually exclusive (with GGX taking precedence) in order to prevent any overlaps in motif assignment. Similarly, the QQ (or SS) motif assignments took precedence over GQ (or GS) in cases where the two patterns juxtapose (*e*.*g*. in a xxGQQxx stretch). Subsequently, the MaSp1 and MaSp2 motif profiles from the 5 reference species were superimposed to generate general consensus profiles that reveal essential features of MaSp1 and MaSp2 tandem repeats encompassing a wide range of taxa. The deduced consensus motif criteria were then cross-validated against the collection of MaSp and MaSp-like sequences found in GenBank encompassing infraorder Araneomorphae, including sequences from cDNA libraries originating from major ampullate silk glands, as well as sequences derived from genomic DNA that have previously been classified as MaSp1 or MaSp2. Only sequences displaying the canonical MaSp pattern of alternating poly-Ala and Gly-rich regions were used in the analysis.

### RNA-seq data analysis

The NCBI Sequence Read Archive (SRA) [35] was queried for transcripts resembling MaSp sequences that are expressed in *Tetragnatha* species. Six separate datasets were identified, which represent mRNA reads from whole-body RNA-seq libraries, as described [36]: *T*. *kauaiensis*, maroon ecomorph (TKM; NCBI accession SRX559918), *T*. *kauaiensis*, green ecomorph (TKG; SRX612477), *T*. *perreirai-*1 (TP1; SRX559940), *T*. *perreirai-*2 (TP2; SRX612486), *T*. *tantalus* (TT; SRX612466), and *T*. *polychromata* green (TPG; SRX612485).

The RNA-seq raw data were converted to FastQ format using SRA-tools (fastq-dump). *De novo* transcriptome assembly was performed using Bridger r2014-12-01 [37], with k-mer = 31 for each of the datasets. MaSp-like sequences were screened by running BLASTX (*E* value < 1^−15^) against the available amino acid sequences in GenBank. Six-frame translations of the MaSp-like sequences were used to identify patterns that conform to the spidroin repetitive or terminal domain sequences.

### Sequence alignments and phylogenetic reconstruction

Amino acid sequences were aligned with Clustal Omega [38] using default parameters, followed by manual adjustments. Phylogenetic relationships among the *Tetragnatha* CTD sequences were calculated by maximum likelihood inference using the JTT+F+G model with 500 bootstrap replicates, as implemented in MEGA 7 [39].

## Results

### Identification of conserved MaSp1 and MaSp2 motifs

The primary stage of the analysis involved the identification of MaSp1 and MaSp2 short motifs that are conserved among the different spider taxa. The approach involved the analysis and comparison of tandem repeat motif compositions from a diverse set of spider species. Five reference species were selected, spanning four spider families: *Latrodectus hesperus* (Theridiidae), *Nephila clavipes* (Nephilidae), *Araneus diadematu*s (Araneidae), *Argiope bruennichi* (Araneidae), and *Euprosthenops australis* (Pisauridae). To ensure reliability, only long sequences originating from major ampullate gland cDNA libraries were included in the primary analysis; the shortest sequence used, ADF4 from *A*. *diadematus*, contained 302 residues in the reported repetitive sequence, and contained eight complete tandem repeats. Sequences used in the preliminary analysis have been established as MaSp1 or MaSp2 based on the presence of proline residues [6, 11, 40, 41]. However, the two dragline spidroins from *A*. *diadematus* (ADF3 and ADF4) were ambiguous in that both were rich in prolines, despite having divergent motif compositions [5]. Based on the reported differential behavior of the purified proteins [42], however, ADF3 and ADF4 were designated as MaSp2 and MaSp1 homologs, respectively; the validity of this classification was also strongly supported by the evaluation of motif arrangements within family Araneidae (see separate section below). Accession codes for all sequences used in this study, as well as other details, are given in S1 Table.

**Fig 1:**
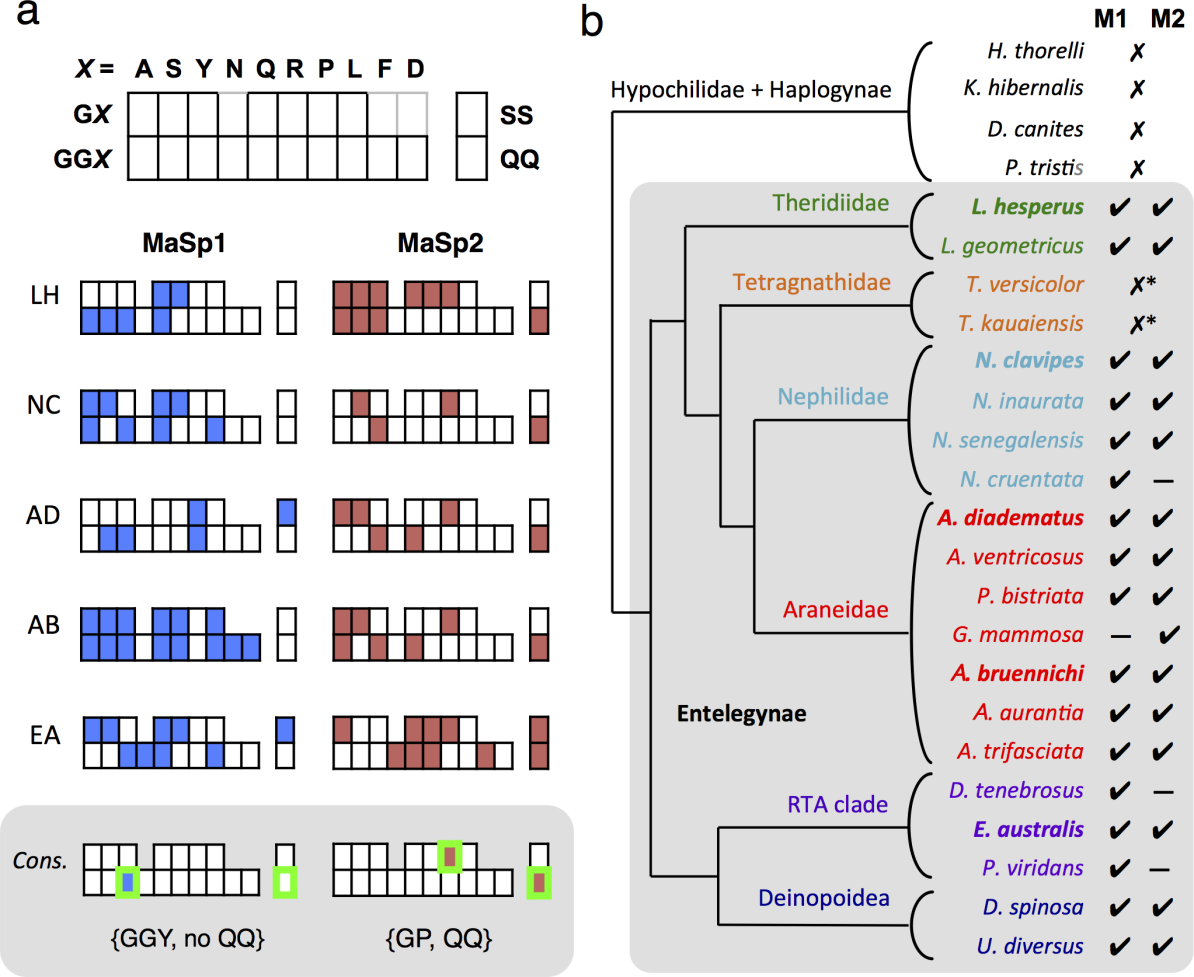
Motif-based analysis of MaSp1 and MaSp2 repetitive regions. (a) Conserved motif profiles of MaSp1 and MaSp2 tandem repeats from five reference species (LH, *Latrodectus hesperus*, NC, *Nephila clavipes*, AD, *Araneus diadematus*, AB, *Argiope bruennichi*, EA, *Euprosthenops australis*). The results are tabulated according to the scheme shown on top, and the resultant consensus motif profiles for MaSp1 and MaSp2 are shown on the bottom, shaded in grey. The results show that all surveyed MaSp1 sequences contain the GGY motif while the MaSp2 sequences all contained GP; furthermore, all of the MaSp2 sequences, but none of the MaSp1, harbored the QQ motif. (b) Analysis of the MaSp sequences recovered from GenBank based on motif profiles. The cladogram, depicted with arbitrary branch lengths, is based on Bond *et al*. [33]. In the rightmost two columns, check marks indicate the presence of sequences that are consistent with the consensus MaSp1 (M1) or MaSp2 (M2) criteria, as outlined in Fig 1A. The two reported sequences from Tetragnathidae showed ambiguous designations based on motif composition, and are marked with asterisks. Species that were used for the initial assessment of motif profiles are indicated in bold, and the Entelegynae clade is shaded in grey. GenBank accessions are listed in S1 Table. LG, *Latrodectus geometricus* (Theridiidae; GenBank AF350273 and AF350274); AV, *Araneus ventricosus* (Araneidae; JN857964 and AB829892); NI, *Nephila inaurata madagascarensis* (Nephilidae; AF350277 and AF350278); UD, *Uloborus diversus* (Uloboridae; DQ399331 and DQ399335); EA, *Euprosthenops australis* (Pisauridae; AJ973155 and AM490169).

Motif profiles were constructed for the MaSp1 and MaSp2 repeat sequences from the five reference species (Fig 1A). Internally aligned tandem repeats were evaluated for regularly appearing motifs, according to the patterns GX and GGX (where X = Ala (A), Ser (S), Tyr (Y), Asn (N), Gln (Q), Arg (R), Pro (P), Leu (L), Phe (F) or Asp (D)). GX and GGX motifs were considered separately as they are thought to adopt different types of conformations in the silk fiber [13, 43, 44]. In addition, the di-serine (SS) and di-glutamine (QQ) doublet motifs were included in the analysis, based on their relatively frequent occurrence. Comparing the conserved motif profiles across the species enabled the derivation of the consensus motif profiles, which revealed some consistent patterns. The GGY motif was observed to be very widely represented in the reference sequences, and emerged as the only conserved motif among all the MaSp1 sequences. GGY was also nearly ubiquitous in MaSp2, being present in four of the five reference sequences (with GGF replacing GGY in *E*. *australis* [11]). On the other hand, the MaSp2 sequences were found to be strictly associated with two motifs, GP and QQ. Intriguingly, none of the MaSp1 reference sequences contained the QQ motif, such that the two MaSp homologs could be distinguished based on this single parameter (Fig 1A).

### Conservation of MaSp1 and MaSp2 motif patterns

To validate the results obtained from the initial analysis, the entire set of MaSp (and MaSp-like) sequences found in GenBank encompassing the true spiders (Araneomorphae) were evaluated on the basis of conserved motif profiles. The analysis yielded remarkably consistent results (Fig 1B). Within the highly diverse Entelegynae, the consensus motif patterns described above could be used to unambiguously differentiate between MaSp1 and MaSp2 sequences in the large majority of cases. The same set of criteria could be applied to sequences from the cribellate orb-weavers (Deinopoidea) and the multi-family RTA clade, even though these groups were only sparsely represented in the preliminary analysis. It should be noted that in some of the species surveyed, only a single type of MaSp homolog is reported; among web building species this is presumed to reflect a lack of available data rather than the absence of particular spidroin expression (*e*.*g*. *N*. *cruentata*, *G*. *mammosa*). However, among some webless spiders (*e*.*g*. *D*. *tenebrosus*, *P*. *viridans*), the absence of MaSp2 data might reflect actual expression patterns, as has been proposed [45, 46]. The accession codes for sequences used in the study are given in S1 Table along with other details.

Conspicuously, the two reported sequences from *Tetragnatha* (Tetragnathidae) displayed motif repertoires that did not conform to the expected MaSp1/MaSp2 profiles, instead showing intermediate characteristics of conserved QQ motifs and the absence of prolines (Fig 1B). Subsequent analysis of *Tetragnatha* RNA-seq data, however, revealed alternative MaSp-like transcripts that were congruent with the consensus MaSp1/MaSp2 profiles (see separate section below).

Outside of Entelegynae, dragline silk sequences from the basal clades that include Hypochilidae and Haplogynae [33] largely did not conform to the MaSp1/MaSp2 consensus motif criteria identified in this study. Instead, the repetitive modules from the basal groups typically featured long and complex arrays of residues, consistent with a non-homologous origin of MaSps between the basal and entelegyne taxa, as proposed [4, 12, 47, 48]. However, one sequence from *H*. *thorelli* (Hypochilidae) bore some similarities to the canonical MaSp pattern, with poly-Ala, (G)GX and QQ motifs (GenBank JX102555) [48].

### Family Araneidae: Repetitive motif arrangements

**Fig 2:**
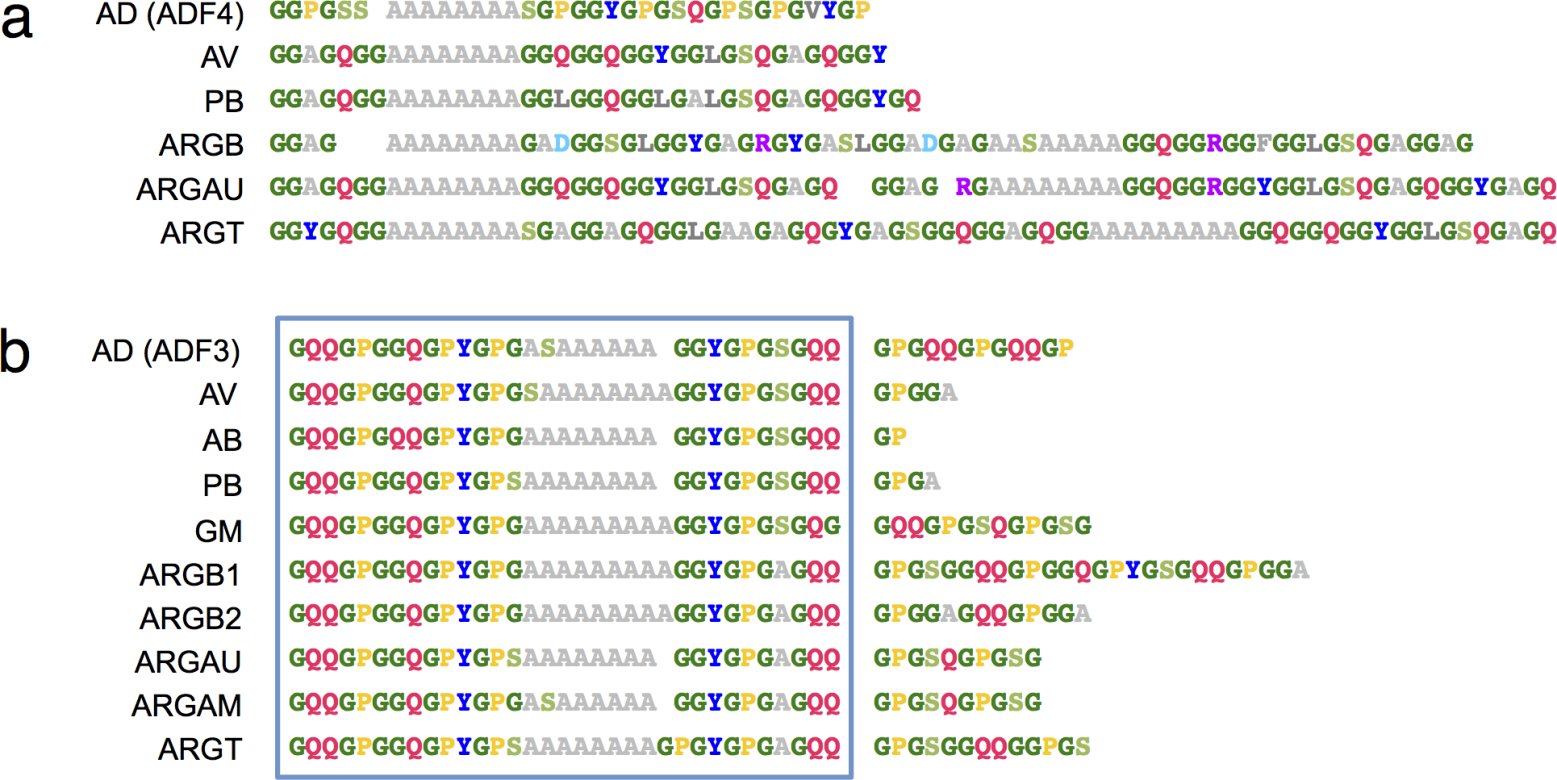
Conservation of tandem repeat motif arrangements within Araneidae. (a) Representative MaSp1 tandem repeat sequences. (b) Representative MaSp2 tandem repeat sequences. A box is drawn around the ~32 residue consensus region. AD, *A*. *diadematus;* AV, *A*. *ventricosus*, AB, *A*. *bicentenarius;* PB, *P*. *bistriata*; GM, *G*. *mammosa*; ARGB, *A*. *bruennichi* (note the two MaSp2 variants), ARGAU, *A*. *aurantia*, ARGAM, *A*. *amoena*, ARGT, *A*. *trifasciata*.

Araneidae comprises the largest family of orb weaving spiders, and the relative abundance of sequence data enabled the comparison of various species and genera from within the group. Strikingly, the MaSp1 and MaSp2 sequences were observed to have very different patterns of sequence conservation (Fig 2). Within each sequence, the MaSp1 homologs displayed highly homogenized internal repeats, however, significant sequence divergence was seen across the different species (Fig 2A). For instance, the *A*. *diadematus* MaSp1 (ADF4) tandem repeats harbored conserved GGY, GP and GS motifs, while the congeneric *A*. *ventricosus* featured GGY, GGQ, GGL, and GGA motifs. The different MaSp1 sequences from *Argiope* all featured a repeat periodicity of two and a highly diverse repertoire of motifs. The patterns observed in the Araneidae MaSp1 repetitive regions are in line with mechanisms of concerted evolution that operate on long and highly repetitive DNA sequences [9, 49, 50].

Strikingly, in contrast to MaSp1, the Araneidae MaSp2 sequences showed a marked degree of interspecific sequence conservation (Fig 2B). A consensus sequences of about 32 residues was identified that corresponds to a major part of the length of each tandem repeat: GQQGPGGQGPYGP(G/S)A_*n*_GGYGPG(A/S)GQQ, with the poly-Ala stretch (A_*n*_) comprising 6–9 contiguous Ala residues (with the occasional inclusion of residues such as Gly, Ser, or Val). In all cases, the poly-Ala region was flanked upstream by a 14-residue section that includes a characteristic GPYGP pattern, and downstream by a 10-residue section that featured the combination GYGPG. Outside the consensus regions are stretches featuring repetitive GP and QQ motifs that show considerable variations in length, even within the same sequence, thus precluding a their reliable alignment. It is emphasized that whereas the repeat sequence of ADF3 matches the deduced MaSp2 consensus sequence, ADF4 clearly does not, despite the abundance of proline residues.

### Tetragnatha: RNA-seq data analysis

MaSp sequences from family Tetragnathidae (long-jawed orb weavers) have so far been reported from only two species, both from genus *Tetragnatha* (*T*. *kauaiensis*, AF350285; *T*. *versicolor*, AF350286). Although the two sequences have been previously classified as MaSp1[4], close evaluation shows that these diverge from the expected MaSp1 pattern by the occurrence of iterated QQ motifs, thus suggesting an intermediate placement between MaSp1 and MaSp2 (Fig 1B). Notably, the two sequences were derived from genomic DNA, instead of gland-specific cDNA libraries, thus the possibility existed that they correspond to non-dragline spidroins. To investigate further, additional *Tetragnatha* spidroin sequences were found by querying the NCBI Sequence Read Archive database [35]; the search yielded six RNA-seq datasets representing whole-body transcriptomes of several closely-related species of *Tetragnatha* [36]. Subsequent contig assembly generated an array of MaSp-like transcript sequences from each dataset, which featured the stereotypical alternations of poly-Ala and Gly-rich regions.

**Fig 3:**
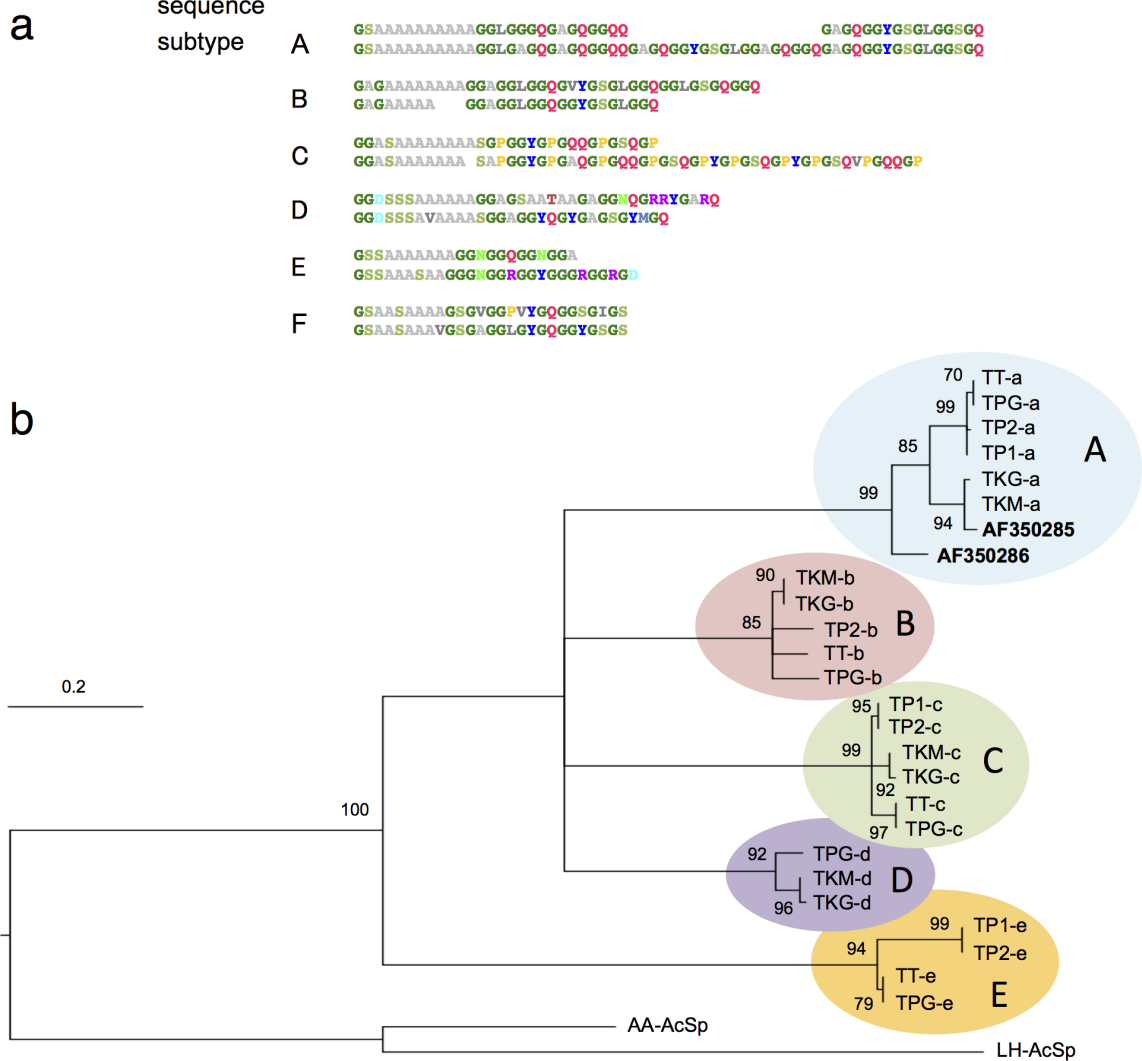
Analysis of *Tetragnatha* MaSp-like sequences derived from RNA-seq libraries. (a) Exemplar tandem repeat sequences corresponding to six distinct MaSp-like subtypes. (b) Phylogenetic reconstruction of the CTD amino acid sequences based on maximum likelihood inference. Posterior support values are indicated; nodes with <60% support value have been collapsed. The tree is midpoint rooted and includes CTD sequences from aciniform spidroin AA-AcSp (*Argiope argentata*; GenBank AHK09813) and LH-AcSp (*L*. *hesperus*; AFX83557). The *Tetragnatha* MaSp-like sequences clustered into five distinct groups in a manner identical to the independent analysis based on the associated tandem repeat sequences (subtypes A-E, in colored ovals). The two original *Tetragnatha* spidroin sequences deposited in GenBank are indicated in bold.

The new *Tetragnatha* MaSp-like sequences shared some similarities in tandem repeat composition, such as the prevalence of poly-Ala, GGY, and GS motifs, but showed apparent differences as well. Based on the motif repertoires of the repetitive domains, these sequences could be classified into distinct groups, denoted MaSp-like subtypes A-F, as illustrated in Fig 3A (with the full set of assembled contigs given in S1 Appendix). In terms of differences, the subtype C sequences, for instance, harbored conserved GS, GGY, GP and QQ motifs (and thus fulfilled the MaSp2 criteria, as deduced in this study). In contrast, the subtypes B, D, and E all had motif repertoires compatible with MaSp1, but featured consistent variations, *e*.*g*. subtype B harbored GGL whereas subtype D was enriched for GGN motifs. The two aforementioned sequences found in GenBank, AF350285 and AF350286, closely matched the MaSp-like subtype A motif repertoire, featuring GS, (G)GQ, GGY, GGL and QQ motifs (but no proline residues) in the tandem repeat regions.

The C-terminal domains (CTD) of the *Tetragnatha* MaSp-like sequences were in many cases recovered along with the tandem repeats, thus providing an additional means of evaluating their relationships. Maximum likelihood analysis of the CTD amino acid sequences yielded a phylogenetic tree that neatly coincided with the classification based on the tandem repeat motifs, strongly supporting the model of the existence of multiple related yet distinct MaSp-like spidroins (Fig 3B). Consistent with MaSp sequences from other taxa [51], the different *Tetragnatha* MaSp-like subtypes all harbored the charged residues R52, D93 (or E93) and E101, as well as the disulfide-forming C92 residue within the CTD (S1 Fig). Variations among the CTDs included the lack of conserved R43 residue in subtypes A and E, which has been shown to participate in salt bridge formation with an acidic residue at position 93 among MaSp and minor ampullate spidroin (MiSp) homologs [51, 52]. N-terminal domains (NTD) were in some cases also recovered from the RNA-seq analysis (S2 Fig). The NTDs from *Tetragnatha* subtypes B and C shared many functionally relevant residues with established MaSp homologs (W10, D39, D40, K60, K65, E79, E84, and E119) [53, 54]. On the other hand, MaSp-like subtype F, while exhibiting conserved features, lacked residues W10 and K65, and is assumed to represent a divergent spidroin variant.

### Relationships between motif abundance and web morphology

**Fig 4:**
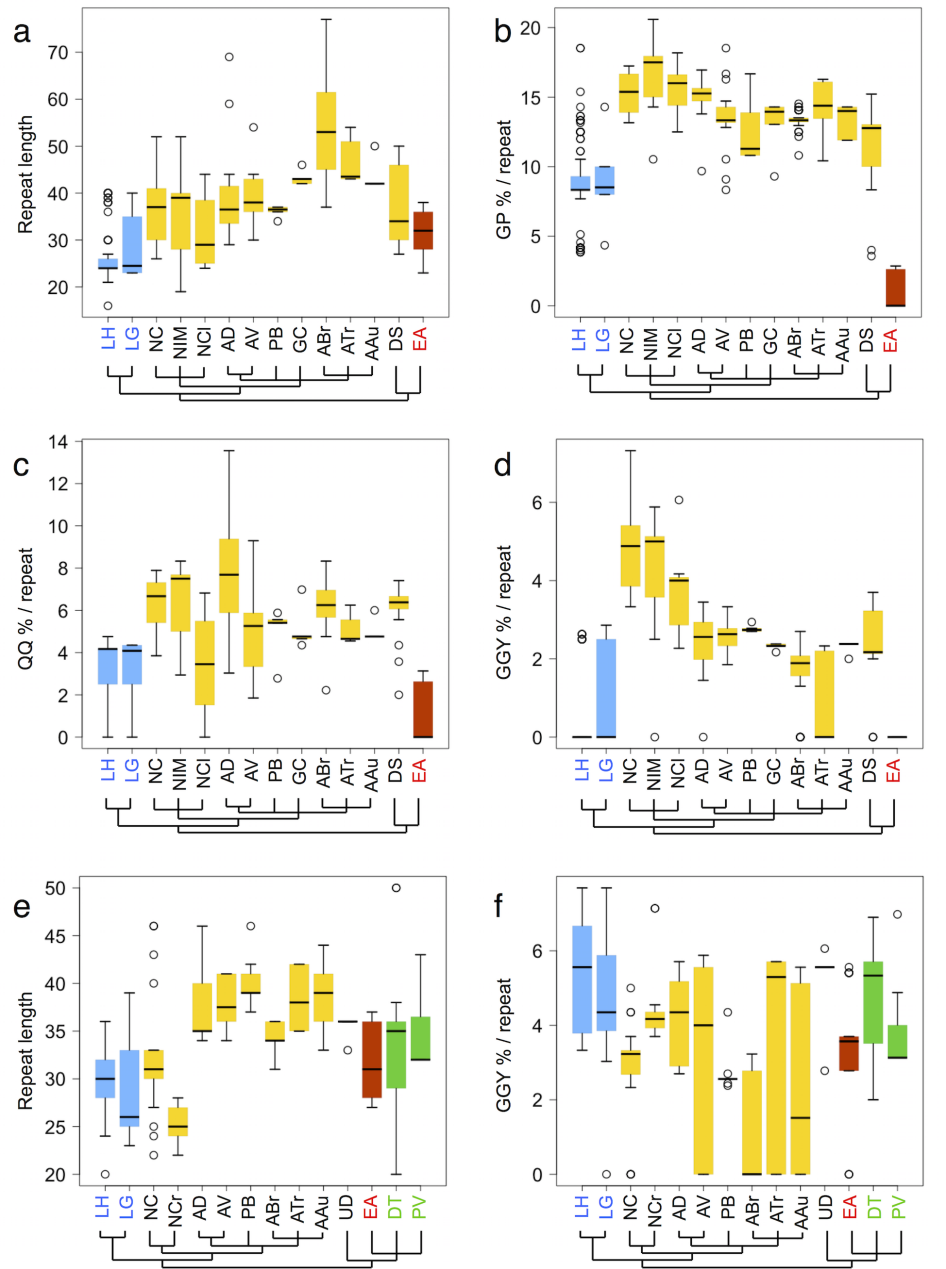
Quantification of conserved MaSp motifs. Box-and-whisker plots show the distribution of repeat lengths and conserved motif prevalence in the repetitive domains of MaSp2 (a-d) and MaSp1 (e-f). Horizontal bars indicate the median value, boxes denote the range of the first and third quartiles, and whiskers represent values within 1.5 times the interquartile range, and circles represent outliers. (a) Tandem repeat lengths in MaSp2 sequences. (b-d) Relative abundance of GP, QQ, and GGY motifs in MaSp2, respectively, calculated as the percent of tandem repeat length. (e) Tandem repeat lengths in MaSp1 sequences. (f) Relative abundance of MaSp1 GGY motif, calculated as the percent of tandem repeat length. Colors indicate the spider web architecture produced by each species: orb web (yellow), three-dimensional cob web (blue), sheet web (red) and no web (green). The cladograms below are based on Bond *et al*. [33], and are shown with arbitrary branch lengths. LH, *Latrodectus hesperus* (GenBank accession DQ409057, EF595245); LG, *Latrodectus geometricus* (AY685201, EU177657); NC, *Nephila clavipes* (M37137, M92913); NIM, *Nephila inaurata madagascarensis* (AF350278); NCla, *Nephila clavata* (AF441245); NCr, *Nephilengys cruentata* (EF638446); AD, *Araneus diadematus* (U47856, U47855); AV, *Araneus ventricosus* (JN857964, AB829892); PB, *Parawixia bistriata* (GQ275359, GQ275359); GC, *Gasteracantha cancriformis* (AF350272); ABr, *Argiope bruennichi* (JX112871, JX202781); ATr, *Argiope trifasciata* (AF350266, AH015065); AAu, *Argiope aurantia* (AF350262, AF350263); UD, *Uloborus diversus* (ABD61596); DS, *Deinopis spinosa* (ABD61594); EA, *Euprosthenops australis* (AJ973155, AM490169); DT, *Dolomedes tenebrosus* (AF350269); PV, *Peucetia viridans* (GU306168).

The MaSp1 and MaSp2 repetitive domain sequences were quantified in terms of repeat lengths as well as the prevalence of the conserved motifs identified in this study (Fig 4 and S2 Table). Tandem repeat lengths exhibited a wide range of variability, with median values for MaSp1 ranging from around 25 to 40 residues (*N*. *cruentata* and *P*. *bistriata*, respectively), while for MaSp2 ranging from around 26 to 52 residues (*L*. *hesperus* and *A*. *bruennichi*, respectively) (Fig 4A and 4E). Intriguingly, analysis of the motif abundance patterns suggests a relationship between the prevalence of MaSp2 motifs (GP, QQ, and GGY) and the type of spider web architecture produced by each species, independent of tandem repeat length (Fig 4A–4D). Overall, the 3 MaSp2 motifs showed the highest prevalence among spiders that construct orb webs (species belonging to Araneidae, Nephilidae, and Deinopoidea), whereas the lowest abundance were observed in the sheet web building *E*. *australis*, with the three-dimensional cobweb building *Latrodectus* displaying intermediate abundance of motifs. For instance, the abundance of the GP motif (calculated as percentage of repeat length) among orb weavers showed median values ranging from 10–17% (reflecting average motif frequencies of >3 per tandem repeat), in cobweb-building *Latrodectus* around 8.5% (average of 2 GP motifs per repeat), while the sheet web building *E*. *australis* exhibited a median GP/repeat value of zero, reflecting an average frequency of 1 GP motif for every 3 tandem repeats. A similar pattern was seen for the abundance of QQ and GGY motifs among the different MaSp2 sequences, despite some outliers (e.g. the relatively low abundance of QQ and GGY motifs in *N*. *clavata* and *A*. *trifasciata* MaSp2 sequences, respectively). As mentioned previously, the MaSp2 repetitive domains of the sheet producing *E*. *australis* were devoid of GGY motifs; Also noteworthy is the fact that no MaSp2 sequences have been reported to date for webless spiders, consistent with the idea that MaSp2 expression is either absent or down-regulated in such species [46].

Since web architecture reflects prey capture strategy and thus implies certain demands on fiber performance (*e*.*g*. aerial orb webs are designed to catch flying insects, reflected in the exceptional toughness values of the component dragline fibers [45, 55], the results of the analysis support the hypothesis that the abundance of the MaSp2 motifs GP, QQ, and GGY play a role in modulating the mechanical properties of dragline silk.

In contrast to the MaSp2 motifs, the conserved GGY motif in MaSp1 showed very high variability in terms of abundance within tandem repeats of the same sequences and across different species, and displayed no obvious relationship with either phylogenetic classification or web architecture (Fig 4E and 4F; S2 Table).

## Discussion

The work described here successfully identified several conserved features of dragline spidroin sequences from a diverse background of non-conserved motif types. In particular, 3 motifs types, GGY, GP, and QQ, appear to hold some significance based on their conservation patterns. From our results, the GGY motif emerged as a near-ubiquitous feature of both MaSp1 and MaSp2 tandem repeats. This suggests that the GGY motif fulfills an important biological function, perhaps analogous to the role of tyrosine in modulating intermolecular self-assembly of silkworm silk [56, 57]. The possibility of di-tyrosine crosslinking in spider dragline silk has also been suggested [58, 59].

MaSp2 sequences were found to be strictly associated with the motifs GP and QQ. Although the requirement for proline is well known, the QQ motif is not generally identified as an essential feature of MaSp2, although the association has been noted in some studies [11, 60]. The role of the QQ motif is unknown; it is likely that its prevalence in MaSp2 repeats does not merely reflect a requirement for elevated Gln levels, since MaSp1 displays a similar abundance, in the form of GQ or GGQ motifs (*e*.*g*. *N*. *clavipes* has a Gln abundance of around 10% and 13% for MaSp1 and MaSp2 tandem repeats, respectively). It is thus likely that the QQ motif *per se* is significant for MaSp2 function.

We speculate that the MaSp2 QQ motifs are relevant for the maintenance of the hierarchical organization of dragline silk. Glutamine-rich polypeptides have a well-known propensity to aggregate via the formation of intermolecular hydrogen bonds, as seen in some β-amyloid fibrils [61–63]. In dragline silk, Gln-Gln hydrogen bond formation might enable intermolecular clustering of MaSp2 molecules, and consequently promote the observed differential localization of MaSp1 and MaSp2 chains in the silk fiber [27, 64], a phenomenon in line with earlier microscopic studies [65, 66].

Intriguingly, the mutual occurrence of conserved proline and QQ motifs was also found to be a prominent feature of pyriform spidroin sequences (constituents of web attachment discs), albeit within different sequence contexts [67, 68]. Moreover, the high molecular weight subunit of glutenin from wheat, responsible for the strength and elasticity of bread dough, also features a highly repetitive central domain that is extremely rich in proline residues and QQ motifs, reminiscent of MaSp2. The glutenin repeats are predicted to adopt flexible β-spiral conformations [69], and structural studies suggest that the prolines provide molecular chain mobility while the glutamine residues participate in an extensive network of intermolecular hydrogen bonds [70, 71].

Our analysis also uncovered an extended, highly conserved arrangement of motifs in MaSp2 tandem repeats from Araneidae, the most successful family of orb weaving spiders. This was surprising, since MaSp motif replacements and rearrangements are common in other spider groups, even at the genus level (*e*.*g*. among *Latrodectus* or *Nephila* sequences). The findings offer some insights into the identities of the two archetypal dragline spidroins from *A*. *diadematus*, ADF3 and ADF4 [5], whose high proline content have led some studies to designate both as MaSp2 variants [4, 60]. Here we provide strong support for ADF3 being a true homolog of MaSp2 based on motif composition, and by virtue of its conformity to the MaSp2 tandem repeat organization within Araneidae. In contrast, ADF4 exhibits a motif composition and arrangement that is clearly divergent from the consensus MaSp2 pattern. Our analysis thus suggests that ADF4 could be a MaSp1 variant that harbors an unusual abundance of proline residues. It should be noted that different Araneidae orb-weaving species (including *A*. *diadematus*) produce dragline silks with comparable material properties despite having highly dissimilar MaSp1 sequences [25, 72], suggesting that the MaSp1 tandem repeats can accommodate relatively large variations in motif composition without sacrificing fiber performance.

RNA-seq data analysis revealed an expanded array of MaSp-like spidroins in genus *Tetragnatha*, all of which bear the stereotypical poly-Ala/Gly-rich features, but otherwise vary in terms of motif composition and organization. Although the short read lengths limited the repetitive domain segments that could reliably be assembled, the remarkable agreement of results from six independent datasets provides compelling evidence for the validity of the approach, a conclusion further supported by the analysis of CTD sequences. The significance of the multiple MaSp-like subtypes, although recent findings based on transcriptomic and proteomic analyses on other spider groups have likewise revealed complex patterns of spidroin expression [73, 74].

The study has several limitations that should be raised. Dragline silk is a composite of MaSp1 and MaSp2; however, the effect of different ratios of MaSp1/MaSp2 in the fiber, which can vary considerably even among individuals of the same species, is beyond the scope of the present study. Another issue is that the quantification of motif prevalence is limited by sequence data quality—in certain cases only short reads, with few repeats, are available, possibly skewing the apparent abundance values. Furthermore, currently not all spider families are represented in the sequence databases. It is hoped that future deposition of high quality sequence data would lead to expanded analyses and novel sequence-property insights.

## Conclusions

In this study, we report the conserved amino acid motifs associated with spider dragline spidroins MaSp1 and MaSp2 across a wide range of spider taxa. The apparent co-expansion of the MaSp2 motifs GP, QQ, and GGY with spiders' prey capture strategy suggests that these motifs play a critical role in modulating the mechanical properties of dragline silk. From a practical standpoint, our results suggest novel, testable hypotheses that can inform future directions in experimental research and should be helpful in efforts to synthesize biomimetic artificial spider silk, or in the design of silk-like biopolymers having customized properties, such as enhanced toughness or water resistance, toward a variety of real-world applications.

## Supporting information

S1 Table Major ampullate spidroin (MaSp) and MaSp-like sequences from GenBank used in this study. (DOC)

S2 Table Relationships between web architecture and repetitive domain sequence features. Mean and median values of repeat length and relative abundance of conserved amino acid motifs in the tandem repeat regions of MaSp1 and MaSp2 sequences. *n* refers to the number of tandem repeats used in each analysis.
(DOCX)

S1 Appendix Assembled MaSp-like contig sequences from *Tetragnatha*, generated from analysis of RNA-seq data from the NCBI-SRA database. Data are arranged according to separate datasets (1–6) and annotated according to the MaSp-like sequence subtype (a-f) as outlined in the main text.
(DOCX)

S1 Fig Aligned C-terminal domains of MaSp-like sequences from *Tetragnatha* RNA-seq data. The sequences have been grouped according to the five MaSp-like subtypes (A-E) as discussed in the main text; sequences obtained from RNA-seq libraries are labeled in blue, while the original sequences from GenBank are labeled in red: TK (*T*. *kauaiensis*; AF350285) and TV (*T*. *versicolor*; AF350285). In addition, MaSp1 (XX-M1) and MaSp2 (XX-M2) sequences from four other families are shown, labeled in black: LH, *Latrodectus hesperus*; AV, *Araneus ventricosus*; NC, *Nephila clavipes*; EA, *Euprosthenops australis*. The locations of the five putative α-helices are shown (H1-H5), and conserved, functionally relevant residues are indicated with asterisks.
(PNG)

S2 Fig Aligned N-terminal domains of MaSp-like sequences from *Tetragnatha* RNA-seq data. Shown are MaSp-like sequences from *Tetragnatha* obtained by RNA-seq analysis (labeled in blue), as well as from other spider families (in black). NTD sequences corresponding to *Tetragnatha* MaSp-like subtypes B, D, and D are shown. MaSp1 (XX-M1) and MaSp2 (XX-M2) from four other species are shown: LH, *Latrodectus hesperus*; NC, *Nephila clavipes*; EA, *Euprosthenops australis*; and AB, *Argiope bruennichi*. Predicted locations of the signal peptide and the five NTD α-helices (H1-H5) are shown, as well as conserved residues important for NTD function (asterisks).
(PNG)
