# Supplementary material for: Analysis of repetitive amino acid motifs reveals the essential features of spider dragline silk proteins
Source: PLoS One. 2017 Aug 23;12(8):e0183397. doi: 10.1371/journal.pone.0183397 (PMC5568437; doi:10.1371/journal.pone.0183397)
Supplement: S1 Table — (DOC) [file pone.0183397.s001.doc]

**Analysis of repetitive amino acid motifs reveals essential features of spider dragline silk proteins**

Ali D. Malay, Kazuharu Arakawa, and Keiji Numata

**Supporting Table S1.** Major ampullate spidroin (MaSp) and MaSp-like sequences from GenBank used in this study.

| **GenBank ID** | **Species name** | **Family** | **Prelim** | **MA gland** | **A*_n_*** | **GGY** | **GP** | **QQ** | **MaSp**  **1** | **MaSp**  **2** | **Other MaSp-like** | **Notes** |
| --- | --- | --- | --- | --- | --- | --- | --- | --- | --- | --- | --- | --- |
| **JX102555** | *Hypochilus thorelli* | Hypochilidae |  | x | • |  |  | • |  |  | • | cDNA from mixed glands |
| **JX102556** | *Hypochilus thorelli* | Hypochilidae |  | x |  |  |  |  |  |  |  | cDNA from mixed glands |
| **HM752564** | *Diguetia canities* | Haplogynae |  | • | • |  |  | ? |  |  | • | large ampullate gland |
| **HM752565** | *Diguetia canities* | Haplogynae |  | • | • |  |  | • |  |  | • | large ampullate gland |
| **HM752567** | *Diguetia canities* | Haplogynae |  | • | • |  |  |  |  |  | • | large ampullate gland |
| **AF350282** | *Plectreurys tristis* | Haplogynae |  | • | • |  | • |  |  |  | • | larger ampullate gland |
| **AF350281** | *Plectreurys tristis* | Haplogynae |  | • | • |  |  |  |  |  | • | larger ampullate gland |
| **AF350283** | *Plectreurys tristis* | Haplogynae |  | x | • |  |  | • |  |  |  | smaller ampullate gland |
| **AF350284** | *Plectreurys tristis* | Haplogynae |  | x | ? |  | • | • |  |  | • | smaller ampullate gland |
| **AY571310** | *Kukulcania hibernalis* | Haplogynae |  | • |  | • |  | • |  |  |  |  |
| **AY571309** | *Kukulcania hibernalis* | Haplogynae |  | • |  |  |  | • |  |  |  |  |
| **AY571308** | *Kukulcania hibernalis* | Haplogynae |  | • |  | • | • |  |  |  |  |  |
| **HM752563** | *Kukulcania hibernalis* | Haplogynae |  | x |  | • | • |  |  |  |  | mixed ? |
| **KM987233** | *Scytodes thoracica* | Haplogynae |  | x |  |  |  |  |  |  |  | cDNA from mixed glands |
| **KM987234** | *Scytodes thoracica* | Haplogynae |  | x |  |  |  |  |  |  |  | cDNA from mixed glands |
| **EF595246** | *Latrodectus hesperus* | Theridiidae |  |  | • | • |  |  | • |  |  | gDNA |
| **DQ409057** | *Latrodectus hesperus* | Theridiidae | • | • | • | • |  |  | • |  |  |  |
| **EF595245** | *Latrodectus hesperus* | Theridiidae |  |  | • | ~ | • | • |  | • |  | gDNA |
| **DQ409058** | *Latrodectus hesperus* | Theridiidae | • | • | • | ~ | • | • |  | • |  |  |
| **AF350273** | *Latrodectus geometricus* | Theridiidae |  | • | • | • |  |  | • |  |  |  |
| **AF350274** | *Latrodectus geometricus* | Theridiidae |  | • | • | • | • | • |  | • |  |  |
| **AY685201** | *Latrodectus geometricus* | Theridiidae |  | • | • | • | • |  | • |  |  |  |
| **DQ059134** | *Latrodectus geometricus* | Theridiidae |  | x | • | • | • |  | • |  |  | gDNA, but N-blot done |
| **DQ059133** | *Latrodectus geometricus* | Theridiidae |  | x | • | • |  |  | • |  |  | gDNA |
| **AF350275** | *Latrodectus geometricus* | Theridiidae |  | x | • | • | • | • |  | • |  | gDNA |
| **EU177657** | *Latrodectus geometricus* | Theridiidae |  | x | • | • | • | • |  | • |  | gDNA |
| **AF350285** | *Tetragnatha kauaiensis* | Tetragnathidae |  | x | • | • |  | • |  |  | • | gDNA |
| **AF350286** | *Tetragnatha versicolor* | Tetragnathidae |  | x | • | • |  | • |  |  | • | gDNA |
| **M37137** | *Nephila clavipes* | Nephilidae | • | • | • | • |  |  | • |  |  |  |
| **U20329** | *Nephila clavipes* | Nephilidae | • | • | • | • |  |  | • |  |  |  |
| **U37520** | *Nephila clavipes* | Nephilidae | • | • | • | • |  |  | • |  |  |  |
| **M92913** | *Nephila clavipes* | Nephilidae | • | • | • | • | • | • |  | • |  |  |
| **AF350277** | *Nephila inaurata m.* | Nephilidae |  | x | • | • |  |  | • |  |  | gDNA |
| **AF350276** | *Nephila inaurata m.* | Nephilidae |  | x | • | • | • | • |  | • |  | gDNA |
| **AF350278** | *Nephila inaurata m.* | Nephilidae |  | x | • | • | • | • |  | • |  | gDNA |
| **DQ059135** | *Nephila inaurata m.* | Nephilidae |  | x | • | • | • | • |  | • |  | gDNA |
| **AF350279** | *Nephila senegalensis* | Nephilidae |  | x | • | • |  |  | • |  |  | gDNA |
| **AF350280** | *Nephila senegalensis* | Nephilidae |  | x | • | • | • | • |  | • |  | gDNA |
| **AY666076** | *Nephila pilipes* | Nephilidae |  | x | • | • |  |  | • |  |  | total cDNA |
| **AF441245** | *Nephila clavata* | Nephilidae |  | • | • | • | • | • |  | • |  |  |
| **ABC72644** | *Nephila antipodiana* | Nephilidae |  | x | • | • |  |  | • |  |  | cDNA from mixed glands |
| **EF638446** | *Nephilengys cruentata* | Nephilidae |  | • | • | • |  |  | • |  |  |  |
| **U47856** | *Araneus diadematus* | Araneidae | • | • | • | • | • |  | • |  |  | ADF-4 |
| **U47855** | *Araneus diadematus* | Araneidae | • | • | • | • | • | • |  | • |  | ADF-3 |
| **JN857964** | *Araneus ventricosus* | Araneidae |  | • | • | • |  |  | • |  |  |  |
| **AB829892** | *Araneus ventricosus* | Araneidae |  | • | • | • | • | • |  | • |  |  |
| **U20328** | *Araneus bicentenarius* | Araneidae |  | x | • | • | • | • |  | • |  | gDNA |
| **GQ275359** | *Parawixia bistriata* | Araneidae |  | • | • | • |  |  | • |  |  |  |
| **GQ275360** | *Parawixia bistriata* | Araneidae |  | • | • | • | • | • |  | • |  |  |
| **AF350272** | *Gasteracantha mammosa* | Araneidae |  | x | • | • | • | • |  | • |  | gDNA |
| **KF032719** | *Cyrtophora moluccensis* | Araneidae |  | x |  |  |  |  |  |  |  | gDNA; non-repetitive central domain; not included in analysis |
| **JX112871** | *Argiope bruennichi* | Araneidae | • | • | • | • |  |  | • |  |  |  |
| **JX112872** | *Argiope bruennichi* | Araneidae | • | • | • | • | • | • |  | • |  |  |
| **JX202781** | *Argiope bruennichi* | Araneidae | • | • | • | • | • | • |  | • |  |  |
| **AF350266** | *Argiope trifasciata* | Araneidae |  | • | • | • |  |  | • |  |  |  |
| **AF350267** | *Argiope trifasciata* | Araneidae |  | • | • | • | • | • |  | • |  |  |
| **AF350268** | *Argiope trifasciata* | Araneidae |  | x | • | • | • | • |  | • |  | gDNA |
| **AH015065** | *Argiope trifasciata* | Araneidae |  | x | • | ~ | • | • |  | • |  | gDNA |
| **AF350262** | *Argiope aurantia* | Araneidae |  | x | • | • |  |  | • |  |  | gDNA |
| **AF350263** | *Argiope aurantia* | Araneidae |  | x | • | • | • | • |  | • |  | gDNA |
| **AY365020** | *Argiope amoena* | Araneidae |  | ? | • | • | • | • |  | • |  |  |
| **ABD61588** | *Deinopis spinosa* | Deinopoidea |  | x | • | • | • |  | • |  |  | combined silk gland cDNA |
| **ABD61593** | *Deinopis spinosa* | Deinopoidea |  | x | • | • | • | • |  | • |  | combined silk gland cDNA |
| **ABD61594** | *Deinopis spinosa* | Deinopoidea |  | x | • | • | • | • |  | • |  | combined silk gland cDNA |
| **ABD61591** | *Deinopis spinosa* | Deinopoidea |  | x |  |  |  |  |  |  |  |  |
| **ABD61592** | *Deinopis spinosa* | Deinopoidea |  | x |  |  |  |  |  |  |  |  |
| **ABD61596** | *Uloborus diversus* | Deinopoidea |  | x | • | • |  |  | • |  |  | combined silk gland cDNA |
| **ABD61600** | *Uloborus diversus* | Deinopoidea |  | x | • | • | • | • |  | • |  | combined silk gland cDNA |
| **AJ973155** | *Euprosthenops australis* | Pisauridae  (RTA) | • | • | • | • |  |  | • |  |  |  |
| **AM490182** | *Euprosthenops australis* | Pisauridae  (RTA) | • | • | • | • |  |  | • |  |  |  |
| **AM490170** | *Euprosthenops australis* | Pisauridae  (RTA) | • | • | • | • |  |  | • |  |  |  |
| **AM490183** | *Euprosthenops australis* | Pisauridae  (RTA) | • | • | • | • |  |  | • |  |  |  |
| **AM490172** | *Euprosthenops australis* | Pisauridae  (RTA) | • | • | • | • |  |  | • |  |  |  |
| **AM490169** | *Euprosthenops australis* | Pisauridae  (RTA) | • | • | • |  | • | • |  | • |  |  |
| **AM490192** | *Euprosthenops australis* | Pisauridae  (RTA) | • | • | • |  | • | • |  | • |  |  |
| **AF350270** | *Dolomedes tenebrosus* | Pisauridae  (RTA) |  | • | • | • |  |  | • |  |  | cDNA from "ampullate gland" |
| **AF350269** | *Dolomedes tenebrosus* | Pisauridae  (RTA) |  | • | • | • |  |  | • |  |  | cDNA from "ampullate gland" |
| **GU306168** | *Peucetia viridans* | Oxyopidae  (RTA) |  | ? | • | • |  |  | • |  |  |  |
| **AY566305** | *Agelenopsis aperta* | Agelenidae  (RTA) |  | • |  |  |  |  |  | • |  | divergent repeat sequence (GX)_n_; not included in analysis |
| **HM752573** | *Agelenopsis aperta* | Agelenidae  (RTA) |  | • |  |  |  |  |  | • |  | divergent repeat sequence (GX)_n_; not included in analysis |

**Additional notes:**

(1) Classification of the spidroin sequences as MaSp1 or MaSp2 (columns 10 and 11) is as described in the main text. "Other MaSp-like" (column 12) corresponds to divergent sequences or those with intermediate features between MaSp1 and MaSp2

(2) Cells marked in column 4 (Prelim) indicate sequences from the reference species that were used in the preliminary motif profile analysis (Fig. 1a)

(3) Cells marked in column 5 (MA gland) indicates sequences derived from mRNA from the major ampullate gland

(4) "gDNA" indicates sequences derived from genomic DNA sequencing experiments
