# Supplementary material for: Analysis of repetitive amino acid motifs reveals the essential features of spider dragline silk proteins
Source: PLoS One. 2017 Aug 23;12(8):e0183397. doi: 10.1371/journal.pone.0183397 (PMC5568437; doi:10.1371/journal.pone.0183397)
Supplement: S2 Table — Mean and median values of repeat length and relative abundance of conserved amino acid motifs in the tandem repeat regions of MaSp1 and MaSp2 sequences. n refers to the number of tandem repeats used in each analysis. (DOCX) [file pone.0183397.s002.docx]

**Supporting Table S2.** Mean and median values of repeat length and relative abundance of conserved amino acid motifs in the tandem repeat regions of MaSp1 and MaSp2 sequences. *n* refers to the number of tandem repeats used in each analysis.

(A) MaSp1 repeat sequences

| **Species** | **GenBank** | ***n*** | **Repeat length (# residues)** | | | **GGY (%/repeat)** | | |
| --- | --- | --- | --- | --- | --- | --- | --- | --- |
|  | **no.** |  | **Mean** | **± sd** | **Median** | **Mean** | **± sd** | **Median** |
| **LH** | *DQ409057* | 31 | 29.81 | 4.17 | 30.00 | 5.25 | 1.37 | 5.56 |
| **LG** | *AY685201* | 27 | 28.89 | 5.06 | 26.00 | 4.71 | 1.53 | 4.35 |
| **NC** | *M37137* | 19 | 32.63 | 6.77 | 31.00 | 2.72 | 1.56 | 3.23 |
| **NCr** | *EF638446* | 15 | 25.13 | 1.96 | 25.00 | 4.48 | 1.11 | 4.17 |
| **AD** | *U47856* | 7 | 37.86 | 4.71 | 35.00 | 4.13 | 1.32 | 4.35 |
| **AV** | *JN857964* | 6 | 37.83 | 2.93 | 37.50 | 3.24 | 2.81 | 4.00 |
| **PB** | *GQ275359* | 10 | 40.00 | 2.49 | 39.00 | 2.73 | 0.58 | 2.56 |
| **ABr** | *JX112871* | 24 | 34.79 | 1.28 | 34.00 | 1.29 | 1.44 | 0.00 |
| **ATr** | *AF350266* | 14 | 38.43 | 3.57 | 38.00 | 3.21 | 2.89 | 5.30 |
| **AAu** | *AF350262* | 10 | 38.50 | 3.63 | 39.00 | 2.20 | 2.47 | 1.52 |
| **UD** | *ABD61596* | 5 | 35.40 | 1.34 | 36.00 | 5.10 | 1.32 | 5.56 |
| **EA** | *AJ973155* | 33 | 31.91 | 4.04 | 31.00 | 3.38 | 1.43 | 3.57 |
| **DT** | *AF350270* | 16 | 33.81 | 8.10 | 35.00 | 4.76 | 1.46 | 5.33 |
| **PV** | *GU306168* | 7 | 34.86 | 4.91 | 32.00 | 3.93 | 1.50 | 3.13 |

(B) MaSp2 repeat sequences

| **Species** | **GenBank** | ***n*** | **Repeat length (# residues)** | | | **GGY (%/repeat)** | | | **GP (%/repeat)** | | | **QQ (%/repeat)** | | |
| --- | --- | --- | --- | --- | --- | --- | --- | --- | --- | --- | --- | --- | --- | --- |
|  | **no.** |  | **Mean** | **± sd** | **Median** | **Mean** | **± sd** | **Median** | **Mean** | **± sd** | **Median** | **Mean** | **± sd** | **Median** |
| **LH** | *EF595245* | 131 | 25.97 | 5.07 | 24.00 | 0.23 | 0.74 | 0.00 | 8.78 | 2.67 | 8.33 | 3.01 | 1.75 | 4.17 |
| **LG** | *EU177657* | 6 | 28.33 | 7.31 | 24.50 | 0.89 | 1.39 | 0.00 | 8.94 | 3.23 | 8.51 | 3.23 | 1.73 | 4.08 |
| **NC** | *M92913* | 13 | 37.00 | 7.80 | 37.00 | 4.86 | 1.11 | 4.88 | 15.19 | 1.46 | 15.38 | 6.27 | 1.36 | 6.67 |
| **NIM** | *AF350278* | 13 | 34.62 | 9.41 | 39.00 | 4.17 | 1.62 | 5.00 | 16.87 | 2.69 | 17.50 | 6.20 | 1.84 | 7.50 |
| **NCl** | *AF441245* | 7 | 32.00 | 8.76 | 29.00 | 3.75 | 1.30 | 4.00 | 15.53 | 1.97 | 16.00 | 3.47 | 2.80 | 3.45 |
| **AD** | *U47855* | 12 | 40.33 | 11.98 | 36.50 | 2.37 | 0.94 | 2.56 | 14.81 | 1.80 | 15.27 | 7.71 | 3.28 | 7.69 |
| **AV** | *AB829892* | 17 | 38.94 | 5.36 | 38.00 | 2.61 | 0.33 | 2.63 | 13.49 | 2.52 | 13.33 | 5.30 | 2.31 | 5.26 |
| **PB** | *GQ275360* | 8 | 36.25 | 1.04 | 36.50 | 2.76 | 0.08 | 2.74 | 12.43 | 2.18 | 11.29 | 5.17 | 0.98 | 5.41 |
| **GC** | *AF350272* | 5 | 43.20 | 1.64 | 43.00 | 2.32 | 0.08 | 2.33 | 12.97 | 2.11 | 13.95 | 5.10 | 1.06 | 4.76 |
| **ABr** | *JX202781* | 51 | 52.37 | 10.08 | 53.00 | 1.74 | 0.74 | 1.89 | 13.34 | 0.59 | 13.33 | 6.33 | 1.12 | 6.25 |
| **ATr** | *AH015065* | 8 | 46.50 | 4.93 | 43.50 | 0.84 | 1.16 | 0.00 | 14.32 | 1.99 | 14.38 | 5.06 | 0.64 | 4.65 |
| **AAu** | *AF350263* | 5 | 43.60 | 3.58 | 42.00 | 2.30 | 0.17 | 2.38 | 13.28 | 1.26 | 14.00 | 5.01 | 0.55 | 4.76 |
| **DS** | *ABD61594* | 21 | 37.76 | 8.17 | 34.00 | 2.33 | 1.13 | 2.17 | 11.37 | 3.09 | 12.77 | 5.99 | 1.24 | 6.38 |
| **EA** | *AM490169* | 15 | 31.47 | 4.97 | 32.00 | 0.00 | 0.00 | 0.00 | 0.90 | 1.32 | 0.00 | 0.92 | 1.35 | 0.00 |
