# Supplementary material for: Analysis of repetitive amino acid motifs reveals the essential features of spider dragline silk proteins
Source: PLoS One. 2017 Aug 23;12(8):e0183397. doi: 10.1371/journal.pone.0183397 (PMC5568437; doi:10.1371/journal.pone.0183397)
Supplement: S1 Appendix — Data are arranged according to separate datasets (1–6) and annotated according to the MaSp-like sequence subtype (a-f) as outlined in the main text. (DOCX) [file pone.0183397.s003.docx]

**S1 Appendix. Assembled MaSp-like contig sequences from *Tetragnatha*, generated from analysis of RNA-seq data from the NCBI-SRA database.** Data are arranged according to separate datasets (1-6) and annotated according to the MaSp-like sequence subtype (a-f) as outlined in the main text.

**(1) *Tetragnatha kauaiensis* maroon (TKM; NCBI accession SRX559918)**

TKM-a

GGLGAGQG

ASAAAAAAAAGGLGGGQGGYGSGLGGVGQGGQGALGGS

RNSATNAISNSASNAVSLLSSPASNARISSAVSALASGAASGPGYLSSVISNVVSQVSSNSGGLVGCDTLVQALLEAAAALVHVLSSSSVGQVNLNTAGYTSQLVGQSVAQAFV

TKM-b

TAAAAAAAGGLGGQGGYGSGQGGQGGYGQGGQGS

AGAAASASATAAASRLSSPAANSRVSSAVSTLASGGASSPAALSSVIGNVVSQVSSSNPGLSGCDILVEALLEVVSALVHVLSSSSVGAVNYSTAGQSTQVVSQSVYQALG

TKM-c

QGPGGTGSQGPGG

ASAAAAAAASAPGGYGPGAQGPGSQGPYGPGSQVPGQQGPG

SSASAAAARLSSPSASSRVSSAASSLVSSGGANSGALSGVISNLVSQISNSSPGLSGCDVLVQALLEVVSALVHILSSSSVGQVNYGASGLSAQLVAQAVAQAVA

TKM-d

AAAAAGGAGGNQGYGASQG

SSAASSVSVSSAASRLSTPEASARVSSAASVLASSGISSPGVLSSVIGDVMSQVGSSGVGLSDCDVMVQSLLEVLSALVHILSSSSVGQVDFGTIGSTAQLVSQAVAEGMVF

**(2) *Tetragnatha kauaiensis* green (TKG; NCBI accession SRX612477)**

TKG-a

AGQGGQQGAGQGGYGSGLGGAGQ

GASAAAAAVAAGGLGGGQGGYGSGLGGVGQGGQGALGGSRNSATNAISNSASNAVSLLSSPASN

ARISSAVSALASGAASGPGYLSSVISNVVSQVSSNSGGLVGCDTLVQALLEAAAALVHVLSSSTVGQVNLNTAGYTSQLVGQSVAQAFV

TKG-b

QGA

GSSAAAAAAAAGGAGGLGGQGGIGSGQGGLGGLGSGQGGQGS

GSAAAAAAAA GGAGGLGGQGGYGSGQGGLGGVGSGQGGQGA

GSAAAAAAAAAGGAGGLGGQGGYGSGQGGQGSGQGGQGGYGQGGQ

GSAGAAASASATAAA

SRLSSPAANSRVSSAVSTLASGGASSPAALSSVIGNVVSQVSSSNPGLSGCDILVEALLEVVSALVHVLSSSSVGAVNYSTAGQSTQVVSQSVYQALG

TKG-c

PGGASAAAAAAAASGPGGYGPGQQGPGSQGP

GGASAAAAAAA SAPGGYGPGAQGPGQQGPGSQGPYGPGSQGPYGPGSQVPGQQGP

ASSASAAAARLSSPSAS

SRVSSAASSLVSSGGANSGALSGVISNLVSQISNSSPGLSGCDVLVQALLEVVSALVHILSSSSVGQVNYGASGLSAQLVAQAVAQAVA

TKG-d

LGGIGSGQGGQGAGS

AAAAAAAGGAGGNRGYGAGRGGYGAGQGGDSSS

AAAAAAAGVAGGDQGYGAGSGYMGQGTGSAAGAAGGAGGNQGYGASQGSSAASSVSVSSAA

SRLSTPEASARVSSAASVLASSGISSPGVLSSVIGDVMSQVGSSGIGLSDCDVMVQSLLEVLSALVHILSSSSVGQVDFGTIGSTAQLVSQAVAEGMVF

TKG-f

MGWSTTATLFLALVSIQAFVVMGNIKLVLQDEKSMDDFMTALTQSMQIYYPFDSDTIDDLFEIKGIVTAGMQKMQSAGKSIDHIINSSKALWISALSEIVSENSVDISIKTDAVTQAMNDAFLQTTGNTDPVTIKEVREMITMFSSLSENDSGSSSSSSVAVSSAGSSGYNAGYSTGGATGVSVSSS

GVGSPGYVQGGYGGGSGS

AASAAAA GSGVGGPVYGQGGSGIGSGS

AASAAAV GSGAGGLGYGQGGYGSGSGG

AAATAAAAGSGAGGPGYGQGGYGQGVLGSGS

GASAAAS GGDGGRGGNGGGRGGGSS

AAAAAGGGN

**(3) *Tetragnatha perreirai* 1  (TP1; NCBI accession SRX559940)**

TP1-a

GQQGAGQGGYGSGLGGTGQGS

AAAAAAAAAAGGLGGGQGAGQGGQQGAGQGGYGSGLGGSGQGS

AAAAAAAAAAGGLGAGQGAGQGGQQGAGQGGYGSGLGGAGQGGQGAGQGGYGSGLGGSGQGS

AAAAAAAAAAGGLGGGQGAGQGGQQGGQGGYGVGQG

AAAAAAAA GGLGGYGSGLGGAGQRGQGALGG

SRNSVTNAVSTSASNAVSLLSSPASNARISSAVSALASGAASSPGSLSSVISNVVSQVSSNSDGLDGCDTLVQALLEVAASLVHVLSSSSVGQVNLSTAGYTSQLVGQSVAQAFM

TP1-b

MSFTSRLALSFLVLFCTQSMFALGQSNSPWSNTQTAESFIRSFLSAVGGSGAFTADQMDDMSSIGDTLMGAMEKMARSNKSSKSKLQALNMAFASSVAEIAVVESGGLSIAAKTNAIADGLSSAFLQTTGAVNRQFINEIRSLITMFAQNNANEVSYGGSSGAGSAASASASAGGYGGGATSSGGYGAGASYGSSGSSSGSVSSSSSGAYGPGPAPQTPSGPAQRGISGPAPQGPSSNGPGPQGPSSSSSVSSSRGPSGYSQGPQGPSGPQGPSAQQGPSSRGPGGYGPG

ASSAAAAAAAAAAAAGGAGGLSGQGQGGLGSEQGGYGSGQGGQGQGGQGAG

ASAAAAAAAAGGAGGLGGQGQGGQ

TP1-c.1

QGPGQQGPGSQGPYGPGSQGPGGEGPGGALPAAAAAPSGPGSQGPGQQGPG

SSASAAAARLSSPSASSRVSSAASSLVSSGAANSGALSGVISNLVSQISNSNPGLLGCDVLVQALLEVVSALVHILSSSSVGQVNYGASGLSAQLVAQAVAQAVA

TP1-c.2

MSFTSRLALSFLVLFCTQSMFALGQSNSPWSNTQTAESFIRSFLSAVGGSGAFTADQMDDMSSIGDTLMGAMEKMARSNKSSKSKLQALNMAFASSVAEIAVVEQGGMSIAVKTNAIADALSSAFLQTTGAVNVQFVNEIRSLITMFAQTNVNEVSYGGGSGGGSAASASASAGGGYGQSPSSGGQAPSYGSSGSSYQTSVGSSSSGGYGPGPQGPGPQQAPQQGSQQQGPGRQGPSAPGPQGPGSQGPSGPGQQGPGSQGPGGPGSQGLGGQGPGG

ASAAAAAAAASGTGGQGPYGPGSQGPGGQGPSGPGSQGPGGQGPYGPQGPGG

ASAAAAAAAASGPGGIGPYGPGSQGPGGQGPYGPGSQGPGGQGPSGPGSQGPG

TP1-e

AAAGGNGGRGGYGGGQGGRENG

SSAAAAAA GGNGGQGGYGGQDGNGGAG

SSAAAAAAAGGNGGQG GNGGAG

SSAAASAAGGGNGGRGGYGGGRGGRGDG

SSAAAAA GGGNGGQGGYGGQDGNGGAG

SSAAAAAAAGGNGGQGGYGRQGGIGGAG

SSAAAAAAAGGNGGQGGYGGGRGGRGDG

SSAAAAA GGGNGGQGGYGGQDGN GG

SSAAAAA GSGDSGRGGYDGGRR GGSG

SSAASAAGGRNGSYGPGNTGGGSISTNQLTSSSTTPRVSSAVSALASGGSWSNEALNGAVSSLMRDVYSSSQGISDCEAMNEVLLELISALVHILSYSSIEYVDYGNVADTSFSVANAFSSAFAY

**(4) *Tetragnatha perreirai* 2  (TP2; NCBI accession SRX612486)**

TP2-a

SAAAAAAAAEA GGLGGGQGAGQGRQQGAGQGAYGSGLGGAGQ

GSAAAAAASAAAAGGLGAGQGAGQGGQQGAGQGGYGSGLGGAGQ

GSAAAAAAAAAA GGLGGGQGAGQGGQQGGQGGYGVGQ

GAAAAAAAA GGLGGYGSGLGGAGQRGQGALGG

SRNSVTNAVSTSASNAVSLLSSPASNARISSAVSALASGAASSPGSLSSVISNVVSQVSSNSDGLDGCDTLVQALLEVAASLVHVLSSSSVGQVNLSTAGYTSQLVGQSVAQAFM

TP2-b.1

GYGSGLGGQGGLGSGKGGQ

GAGAAAAAAAAGGAGGL GGYGSGLGGQGGLGSGQ

GAGAAAAAAAAGGAGGLGGQGGYGSGLGSQGGLGSGQGGQ

GSGAAAAAAAAGGAGGLGGQGGYGSGLGGQGGLGSGQGGQ

GAGAAAAA GGAGGLGGQGGYGS GQGGY GQGGQ

GSAAAAASASATAAA

SRLSSPAASSRVSSAVSTLASGGASSPAALSSVIGSVVSQVSSSSPGLSGCDILVEALLEVVSALVHVLSSASVGTVNYSTAGQSTQMVSQSVYQAMG

TP2-b.2

MSFTSRLALSFLVLFCTQSMFALGQSNSPWSNTQTAESFIRSFLSAVGGSGAFTADQMDDMSSIGDTLMGAMEKMARSNKSSKSKLQALNMAFASSVAEIAVVESGGLSIAAKTNAIADGLSSAFLQTTGAVNRQFINEIRSLITMFAQNNANEVSYGGSSGAGSAASASASAGGYGGGATSSGGYGAGASYGSSGSSSGSVSSSSSGAYGPGPAPQTPSGPAQRGISGPAPQGPSSNGPGPQGPSSSSSVSSSRGPSGYSQGPQGPSGPQGPSAQQGPSSRGPGGYGPGA

SSAAAAAAAAGGAGGLGGQGQGGQGQGGLGSGLGGQGAYGSGQGGQ

GAGAPAAAAAGGAGVQGQGGLGSGLGGQGGYGSGQGGQGQGGQ

GAGASAAAAAAAASGAGGQGQGGLGSGLGGQGGYGSGQGGQ

GAGASAAAAAAAA

TP2-c.1

GGYGPGSQGPGPQGPGSQGPGVQGPYGPGSQGPGQQGPGSQGPYGPGSQGPGGQ

GAGGAAAAAAAASGPGGQGPSGSGSQGPGGSGSQGPYGPGSQGPGQQGPG

SSASAAAARLSSPSASSRVSSAASSLVSSGAANSGALSGVISNLVSQISNSNPGLLGCDVLVQALLEVVSALVHILSSSSVGQVNYGASGLSAQLVAQAVAQAVA

TP2-c.2

EIAVVEQGGMSIAVKTNAIADALSSAFLQTTGAVNVQFVNEIRSLITMFAQTNVNEVSYGGGSGGGSAASASASAGGGYGQSPSSGGQAPSYGSSGSSYQTSVGSSSSGGYGPGPQGPGPQQAPQQGSQQQGPGRQGPSAPGPQGPGSQGPSGPGQQGPGSQGPGGPGSQGLGGQGPGGA

SAAAAAAAAASGPGGQRSYGPGSQGPGGQGPSGPGSQGPGGQG

TP2-e

GNGGAG

SSATASAAGGGNGGQGGYGGQDGNGGAG

SSAAAAAAAGGNGGQGGYGGQGGNGG

SSAAAAAGSGDSGRGGYDGGRRGGSG

SSAASAAGGRNGSYGPGNTGGGSISTNQLTSSSTTPRVSSAVSALASGGSWSNEALNGAVSSLMRDVYSSSQGISDCEAMNEVLLELISALVHILSYSSIEYVDYGNVADTSFSVANAFSSAFAY

TP2-f

MGWSTTAMLFLAVVSIQAFVVMGNMKIVLQDESTMDAFMTTLCQSMQNYYPFDSDTIDDLFEIKGIVTAGMQKMQSAGKSIDHIINSSKALWISALSEIVSENSVDISSKTDAVTQAMTDAFLQTTGRADPVTIKEVREMITMFSSLSENDSGSSSSSSVAVSSAGSSGYNAGYSTGGASGVSVSSSGVGSPGYVQGGYGGRSG

SAASAAAAGSGSGGPGYGQGGYGSGSG

SAASVAAAGSGAGGRGIGQGGYGSGPG

SAASAAAAGTGAGGRGIGQGGYGQGGYGSGSG

GAAASAAGAGGPGYGQGGYSSGSG

GAAATAAAAGSGAGGSGIGQGGYGSG

**(5) *Tetragnatha tantalus* (TT; NCBI accession SRX612466)**

TT-a

AGQ

GAAAAAAAAAAAAGGLGAGQGAGQGGQQGAGQGGYGSGLGGAGQ

GAASAAAAAAAA GGLGGGQGAGQGGQQGAGQGGYGSGLGGAGQ

GSAAAAAAAAAE GGLGSGQGAGQGVQQGAGQGGYGSGLGGAGQ

GAASAAAAAAAA GGLGGGQGAGQGGQQGAGQGGYGSGFGGAGQ

GAASAAAAAAAA GGLGGGQGASQGGQQ GGQGGYGAGQGAAAG

SRNSVTNAVSTSASNAVSLLSSPASNARISSAVSALASGAASSPGSLSSVISNVVSQVSSNSDGLDGCDTLVRALLEVAASLVHVLSSSSVGQVNLSTAGYTSQLVGQSVAQAFM

TT-b

QGGYGSGQGGQGQGGYGTGQGGQ

GAGASAAAAAA GGAGGLGGQGQGGLGSGLGGQGGYGSGQGGQGQGGQ

GAGASAAAAAAAGGSGGLGNQGGYGSGLGGQGGLGS GQGGQ

GAGSAAAAAAA GGAGGLGGQGGYGSGQGGQGGY GQGGQ

GSSASAASASATAAA

SRLSSPAASSRVSSAVSTLASGGASSPAALSSVIGSVVSQVSSSNPGLSGCDILVEALLEVVSALVHVLSSASVGAVNYSTAGQSTQIVSQSVYQAMG

TT-c

QGPGQQGPGSQGPGQQGPYGPQGP

GGASAAAAAAAASGPGGYGPGAQGPGQQGPGSQGPYGPGSQGP

GSSASAAAARLSSPSAS

SRVSSAASSLVSSGAANSGALSGVISNLVSQISNSSPGLSGCDVLVQALLEVVSALVHILSSSSVGQVNYGASGLSAQLVAQAVAQAVA

TT-e

GQGGYGGKDGNGGAGS

SAAAAAAAGGNGGQGGYGGQDGNGGAGS

SAAAASAAGGDGGRGGYGGGRGGRGDGS

SAAASAGGGNGSYGPGNSGGGSIATNQLTSSATT

SRVSTAVSSLASGGSWSNEALNGVVSSLMRDVYASSQGISDCEAMNEVLLELISALVHILSYSSISYVDYGNVGDTSYSVANAFNSASAY

**(6) *Tetragnatha polychromata* green (TPG; NCBI accession SRX612485)**

TPG-a

AGQGGQGAGLGGYGSGLGGAGQ

GAASTAAAAAAAGGLGGGQGAGQGGQQGAGQGGYGSGLGGAGQ

GAASAAAAAAAAGGLGGGQGAGQGGHQGAGQGGYGSGLGGAGQ

GAASAAAAAAAAGGLGGGQGASQGGQQ GGQGGY GAGQ

GAAAG

SRNSVTNAVSTSASNAVSLLSSPASNARISSAVSALASGAASSPGSLSSVISNVVSQVSSNSDGLDGCDTLVRALLEVAASLVHVLSSSSVGQVNLSTAGYTSQLVGQSVAQAFM

TPG-b.1

GLGGQGGYGSGLGGQGRLSSGQGGQ

GAGAAAAAAAAGGAGGLGGQGVYGSGLGGQGGLGSGQGGQ

GAGAAAAA GGAGGLGGQGGYGSGLGGQ

GAGAAAAAAA GGAGGLGGQGGYGSGQGGQ GGYGQGGQ

GSAAAAASASATSAA

SRLSSPAASSRVSSAVSSLASGGASSPAALSNVIGSVVSQVSSSNPGLSGCDILVEALLEVVSALVHVLSSANVGAVNYSTAGQSTQIVSQSVYQAMG

TPG-b.2

MSYTSRLALSFLVLFCTQSMIALGQSNSPWSNIQTAESFIRSFLSAVGGSGAFTADQMDDMSSIGDTLMGAMDKMARSNKSSKSKLQALNMAFASSVAEIAVVESGGLSIAAKTNSIADGLSSAFLQTTGAVNRQFINEIRSLITMFAQNNANEVSY

GGSSGGGSAASASAAAGGYGGGAPSSGGYAAGPSYGP

SGSSSVSVSSSSSGAYGPGPQGPSGPGLQGPSSYGPGPQGP

SSSISISVSSSRGPSGYGPGPQGPSGQQGPSGPQGPSGQQGPSSQGPGGYGP

GASSAGASAAATGGQGGQGAGASAAAAGGAGGLGGQGQGGLGSGLGGQGG

TPG-c.1

GPGSQGPYGPGSQGPGSSASAAAA

RLSSPSASSRVSSAASSLVSSGAANSGALSGVISNLVSQISNSSPGLSGCDVLVQALLEVVSALVHILSSSSVGQVNYGASGLSAQLVAQAVAQAVA

TPG-c.2

MSYTSRLALSFLVLFCTQSMIALGQSNSPWSNIQTAESFIRSFLSAVGGSGAFTADQMDDMSSIGDTLMGAMDKMARSNKSSKSKLQALNMAFASSVAEIAVVESGGLSIAAKTNSIADGLSSAFLQTTGAVNVQFVNEIRSLITMFAQTNVNEVSY

GGGSGGGSAASASASAGGGYGQSQSSGAQAPSYGSSGSSYQTSASSP

SSGGYVPGPQGPGPQQAPQQGPGRQGPSGPGQQGPGSQGPSGPVSQGPSGPGSQGPSGPG

/GPG

GASAAAAAAAASGPGGQGPYGPGSQGPGSQGPS

TPG-d

AAGGAGGDGGNGAGRGGFGAGQGGDS

SSAAAAAAGGAGSAATAAGAGGNQGRRYGARQGGDSS

SAVAAAASGGAGGYQGYGAGSGYMGQGAGLAAGAAGDAGGNQGYGAGQGSS

AASSVSVSSAA

SRLSTPEASARVSSAASVLASSGTSSPGVLSSVIGDVMSQVGSSGSGLSDCDVMVQSLLEVLSALVHILSYSSVGQVDFGAIGFSAQLV

TPG-e

GGQGGNGGAES

SAAAAAAAGGNGGQGGYGGGRGGRGDGS

SAAASAGGGNGSYGPGNSGGGSIATNQLTSSATT

SRVSTAVSSLASGGSWSNEALNGVVSSLMRDVYASSQGISDCEAMNEVLLELISALVHILSYSSISYVDYGNVGDTSYSVANAFNSASAY

TPG-f

MGWSTTAMLFLAVVSIQAFVVMGNMKMVLQDENTMDAFMTTLCQSMQNYYPFDSDTIDDLFEIKGIVTAGMQKMQSAGKSIDHIINSSKALWISALSEIVSENSVDISSKTDAVTQAMTDAFLQTTGRADPVTIKEVREMITMFSSMSENDSGSSSSSSVAVSNAGSSGYNAGYSTGGATGVSVSSSGV

GSPGYVQGGYGGGSGS

AASTAAAGSGAGGGPGYGQGGYGSGSGS

AASAAAAGSGAGE
