# Supplementary figures and images for: Analysis of repetitive amino acid motifs reveals the essential features of spider dragline silk proteins
Source: PLoS One. 2017 Aug 23;12(8):e0183397. doi: 10.1371/journal.pone.0183397 (PMC5568437; doi:10.1371/journal.pone.0183397)

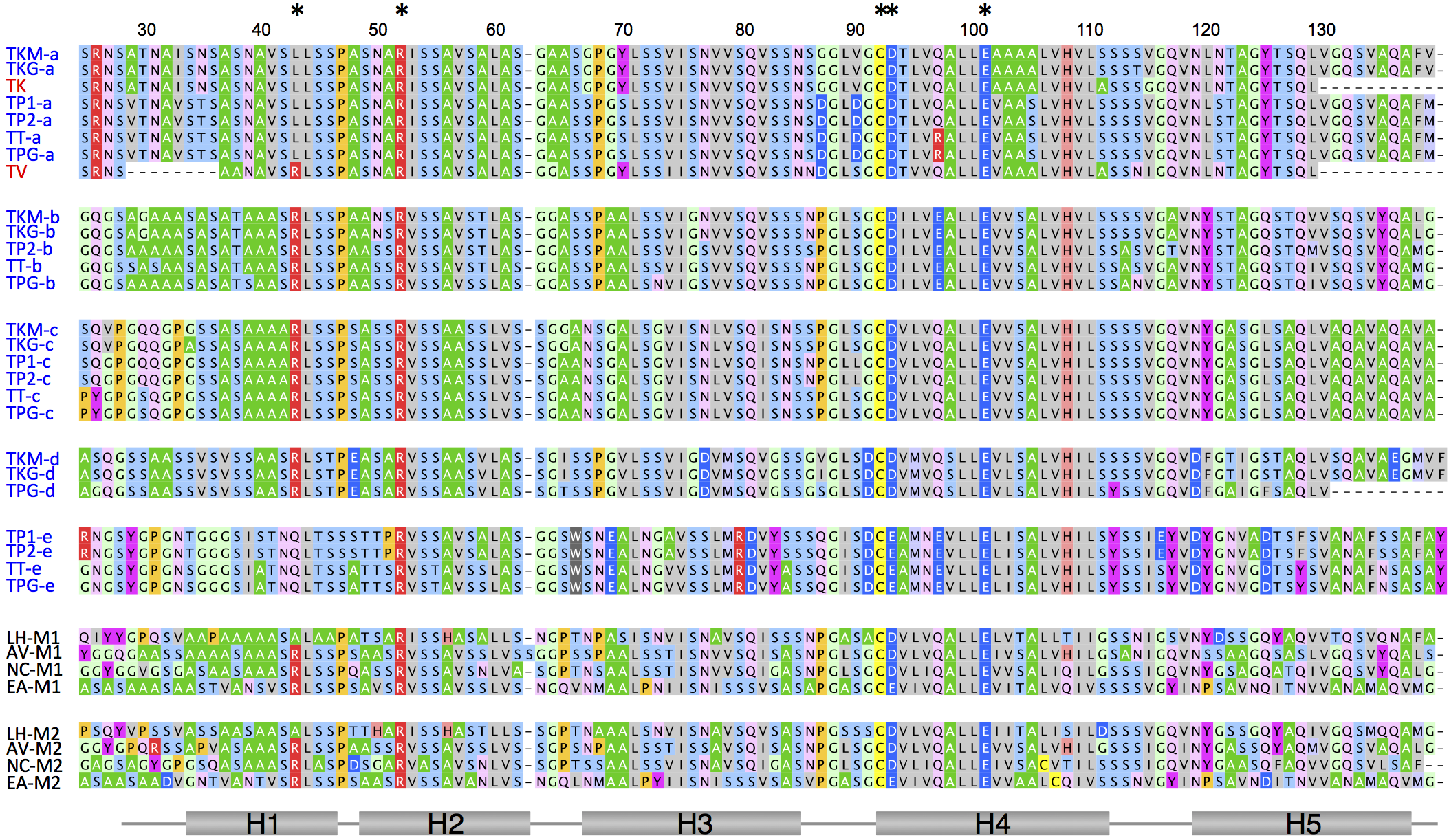

Supplement: S1 Fig — The sequences have been grouped according to the five MaSp-like subtypes (A-E) as discussed in the main text; sequences obtained from RNA-seq libraries are labeled in blue, while the original sequences from GenBank are labeled in red: TK (T. kauaiensis; AF350285) and TV (T. versicolor; AF350285). In addition, MaSp1 (XX-M1) and MaSp2 (XX-M2) sequences from four other families are shown, labeled in black: LH, Latrodectus hesperus; AV, Araneus ventricosus; NC, Nephila clavipes; EA, Euprosthenops australis. The locations of the five putative α-helices are shown (H1-H5), and conserved, functionally relevant residues are indicated with asterisks. (PNG) [file pone.0183397.s004.png]

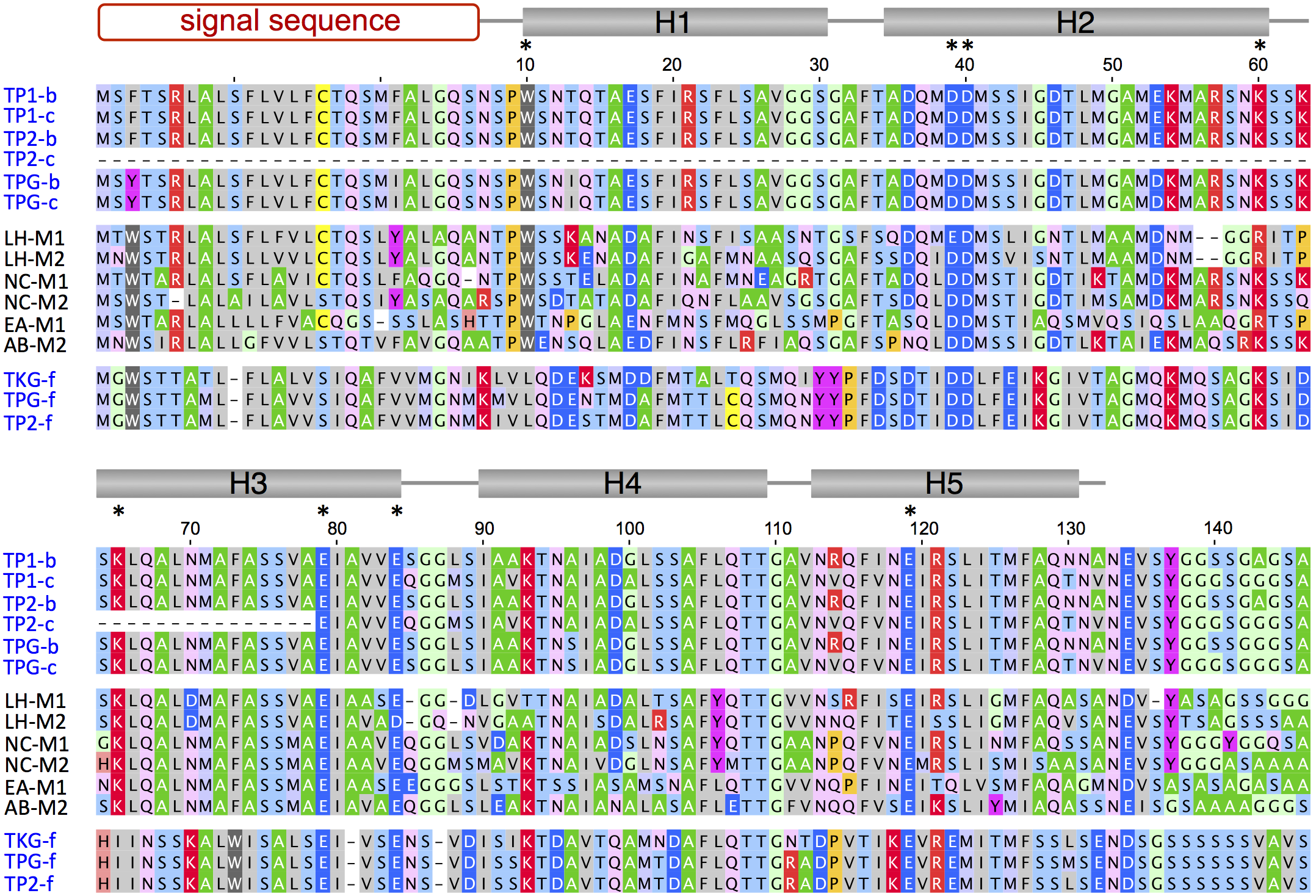

Supplement: S2 Fig — Shown are MaSp-like sequences from Tetragnatha obtained by RNA-seq analysis (labeled in blue), as well as from other spider families (in black). NTD sequences corresponding to Tetragnatha MaSp-like subtypes B, D, and D are shown. MaSp1 (XX-M1) and MaSp2 (XX-M2) from four other species are shown: LH, Latrodectus hesperus; NC, Nephila clavipes; EA, Euprosthenops australis; and AB, Argiope bruennichi. Predicted locations of the signal peptide and the five NTD α-helices (H1-H5) are shown, as well as conserved residues important for NTD function (asterisks). (PNG) [file pone.0183397.s005.png]
